# A cluster randomized controlled trial examining the effects of a four-week mindfulness-based practice on primary school students’ interpersonal mindfulness, emotional intelligence, and attentional focus

**DOI:** 10.3389/fpsyg.2025.1539962

**Published:** 2025-02-25

**Authors:** Li-Jen Lin, Yu-Hsun Lin, Su-Ping Yu, Thu-Hua Liu, Yi-Lang Chen

**Affiliations:** ^1^General Education Center, Ming Chi University of Technology, New Taipei, Taiwan; ^2^Mindfulness Meditation Center, Ming Chi University of Technology, New Taipei, Taiwan; ^3^Department of Business and Management, Ming Chi University of Technology, New Taipei, Taiwan; ^4^Department of Industrial Engineering and Management, Ming Chi University of Technology, New Taipei, Taiwan; ^5^Department of Industrial Design, Ming Chi University of Technology, New Taipei, Taiwan

**Keywords:** mindfulness-based practice (MBP), cluster randomized controlled trial, schoolchildren, interpersonal mindfulness, emotional intelligence, attentional focus

## Abstract

**Background:**

Although mindfulness benefits are well-established across diverse populations, its impact on primary school children’s interpersonal mindfulness, emotional intelligence, and attentional focus remains underexplored. This study evaluated the effectiveness of a four-week mindfulness-based practice (MBP) intervention among primary school students.

**Methods:**

A cluster randomized controlled trial was conducted with 14 classes from Guanghua Elementary School, Taiwan. Classes were randomly assigned to either an intervention group (7 classes, *n* = 123) or a control group (7 classes, *n* = 158), encompassing middle and senior-grade male and female students. The intervention group participated in weekly 40-min MBP sessions over 4 weeks. Students completed three validated questionnaires measuring interpersonal mindfulness dimensions (Presence, PR; Awareness, AW; Nonreactivity, NR), emotional intelligence (EI), and mindfulness (MI, attentional focus), using 6-point Likert scales. Assessments were administered at baseline, immediately after the intervention, and at 8-week follow-up.

**Results:**

Baseline scores revealed no significant differences between the groups across all measures. After the 4-week intervention, the intervention group showed significant improvements in PR (*p* < 0.01), NR (*p* < 0.01), and MI (*p* < 0.001) compared to the control group. At the 8-week follow-up, improvements in PR and MI were maintained, while NR gains diminished. Three-way ANOVA identified that gender and grade significantly influenced AW (*p* < 0.01) and PR (*p* < 0.05), respectively, with stage effects observed for NR and EI (*p* < 0.05). Notably, the intervention enhanced AW among boys, and gender-grade interactions had a significant impact on NR and EI outcomes.

**Conclusion:**

Overall, the four-week MBP intervention significantly enhanced primary school students’ interpersonal mindfulness and attentional focus, with some benefits lasting up to 8 weeks. These findings highlight the importance of tailoring MBP programs to students’ developmental stages and gender-specific needs. Future studies should investigate longer interventions and incorporate objective measures to further validate these outcomes.

## Introduction

Mindfulness, defined as both a dispositional trait and a momentary state of nonjudgmental awareness of thoughts, emotions, and experiences (Williams and Kabat-Zinn, 2013), has gained prominence as a valuable intervention in educational settings through various mindfulness practices (Beshai et al., 2023; Lin et al., 2024). While extensive research highlights mindfulness’s positive effects across diverse populations, its specific influence on primary schoolchildren—particularly in the areas of interpersonal mindfulness, emotional intelligence, and attentional focus—remains underexplored (Porter et al., 2022; Kander et al., 2024). This knowledge gap is particularly critical given the growing mental health challenges and the heightened importance of social–emotional development for young students in today’s educational environments.

The primary school years are a critical phase for children’s social–emotional development and cognitive growth. During this period, children acquire essential interpersonal skills, emotional regulation abilities, and attention control mechanisms, all of which play a pivotal role in their future academic achievement and psychological well-being (Denham et al., 2009; Malti and Noam, 2016; Wolf et al., 2021). Emerging evidence suggests that early mindfulness interventions may support these developmental processes (Zelazo and Lyons, 2012; Bockmann and Yu, 2023), yet comprehensive research in this area remains scarce. Incorporating mindfulness practices into primary education may equip students with effective strategies to navigate the academic demands and social challenges characteristic of this formative stage.

Research on mindfulness-based interventions for primary school children highlights three interrelated developmental constructs that are particularly responsive to mindfulness practices. First, interpersonal mindfulness involves awareness and attentiveness during social interactions, encompassing presence, self/other awareness, and nonreactivity in social contexts (Pratscher et al., 2019). This construct is associated with improved social relationships and conflict resolution skills (Yang et al., 2023), which are especially critical during the primary school years when children establish enduring social patterns and behaviors. Second, emotional intelligence —the ability to recognize, understand, and manage emotions— plays a significant role in academic success and social adaptation (Wong and Law, 2002; Costa and Faria, 2023). Evidence suggests that early intervention can foster long-term improvements in emotional regulation and social competence (Lee et al., 2023). Third, attentional focus, defined as the capacity to sustain voluntary attention on present-moment tasks and experiences (Apaolaza et al., 2019), has been shown to improve through mindfulness practices, with potential benefits for cognitive performance and learning outcomes (Mora Álvarez et al., 2023). Together, these constructs represent interconnected dimensions of child development that are particularly amenable to mindfulness interventions during this formative stage (Maynard et al., 2017).

Several key theoretical frameworks underpin the potential effects of mindfulness interventions on children’s development. Cognitive development theory and neuroplasticity research suggest that while brief mindfulness training can trigger neural changes, sustained practice may be required for enduring benefits (Zelazo and Lyons, 2012). Developmental stage theory posits that children’s cognitive maturation and executive function development affect their ability to engage with and benefit from mindfulness practices, with varying impacts across age groups (Maynard et al., 2017). Social–emotional development literature identifies gender-specific differences in emotional processing and attention regulation, indicating that boys and girls may respond differently to mindfulness training (Yang et al., 2023). Furthermore, cultural-developmental theory highlights the role of sociocultural factors in shaping the acceptance, implementation, and effectiveness of mindfulness programs, particularly within educational settings (Blignault et al., 2023).

Building on these theoretical foundations, several key questions persist regarding mindfulness interventions for primary schoolchildren. Intervention duration remains a critical issue; while some studies report significant benefits from short-term practices (Lim et al., 2023), the long-term sustainability of these effects is underexplored (Volanen et al., 2023). This is particularly pertinent in school settings, where balancing time constraints with intervention effectiveness is a practical challenge. Age-related factors informed by developmental theory further highlight the need for appropriate age adaptations, with evidence suggesting varying effectiveness across age groups (Carsley et al., 2018) and emphasizing the importance of aligning interventions with children’s developing focus and awareness capabilities (Maynard et al., 2017). Additionally, cultural context introduces complexity, especially in Asian educational settings, where cultural attitudes toward meditation and self-reflection may shape intervention outcomes (Blignault et al., 2023; Haidar et al., 2024).

This study fills these gaps through a cluster-randomized controlled trial examining the effects of a four-week mindfulness-based practice (MBP) intervention on primary school children’s development. Specifically, it aims to (1) evaluate the optimal intervention duration by assessing both immediate effects and eight-week retention of MBP benefits on interpersonal mindfulness, emotional intelligence, and attentional focus; (2) investigate developmental stage effects by comparing outcomes between middle-and senior-grade students; (3) explore gender-specific responses to mindfulness training across developmental stages; and (4) assess the cultural adaptation and acceptance of mindfulness practices within Taiwanese schools. Grounded in theoretical frameworks, this study hypothesizes that: (1) the four-week intervention will yield immediate effects, with partial retention at follow-up; (2) senior-grade students will show greater improvements in emotional intelligence than middle-grade students; (3) gender differences will manifest in interpersonal mindfulness outcomes; and (4) the cultural context of Taiwanese education will shape program acceptance and effectiveness.

## Materials and methods

### Participants

**Figure 1:**
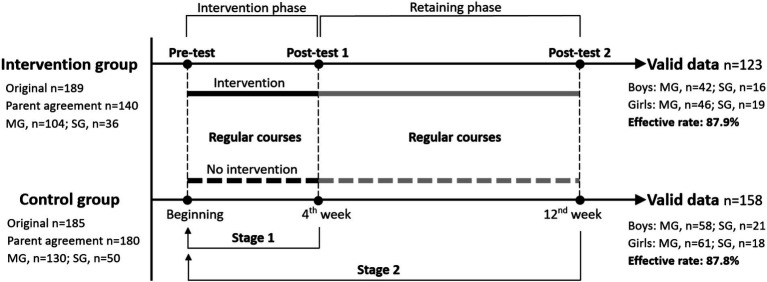
Timeline of the study’s assessment procedure and sample distribution across subgroups at different stages. n = sample size; MG = middle-grade; SG = senior-grade.

The study recruited fourteen classes from Guanghua Elementary School (New Taipei, Taiwan), encompassing middle grades (Grades 3–4) and senior grades (Grades 5–6). Classes were randomly assigned to either the intervention group (*n* = 7 classes) or the control group (*n* = 7 classes), with a total of 374 children participating. The intervention spanned 4 weeks, followed by an eight-week retention period, with assessments conducted at three time points: pre-test, first post-test, and second post-test, as illustrated in Figure 1. A *post-hoc* power analysis using G*Power (version 3.1.9.7) confirmed the sample size was sufficient to detect between-group differences across all dimensions (power = 0.80, *α* = 0.05). A preliminary questionnaire validation was performed with 32 students from the same grades, who were excluded from the main study to prevent contamination. The study protocol was approved by the Ethics Committee of Chang Gung Memorial Hospital, Taiwan (code: 202202334B0), and informed consent was obtained from all participants and their parents.

### Mindfulness-based practice (MBP)

This study implemented a MBP program, supported by the Wang Chang-Gung Charitable Trust Fund, Formosa Plastics Group (WCGCTF, FPG), Taiwan. This organization promotes mindfulness initiatives in local schools as part of its Corporate Social Responsibility (CSR) efforts. The four-week program was designed by the first author, Dr. L.-J. Lin, a certified Mindfulness-Based Cognitive Therapy for Life (MBCT-L) teacher (certified October 2020) and former M1–M4 Team Supervisor (April 2016–March 2019) at the University of Oxford’s Mindfulness Centre.

Following the recommendations of Maynard et al. (2017), Dr. Lin adapted the MBCT-L framework to create an age-appropriate program for schoolchildren, ensuring the retention of its core structure and techniques. The modified MBP focused on three key areas: interpersonal mindfulness, emotional intelligence, and attentional focus. Over 4 weeks, students participated in weekly 40-min sessions, beginning with an introduction to mindfulness in the first week, followed by three specialized units: “Mindfulness for Good Relationships,” “Mastering Emotions,” and “Mindfulness in Learning.” The sessions incorporated diverse practices such as gratitude exercises, mindful listening, meditation, and deep breathing to engage the students effectively.

### Questionnaire development

Three questionnaires were used to evaluate the impact of the MBP intervention: Interpersonal Mindfulness (IM), Emotional Intelligence (EI), and Mindfulness (MI) (complete versions available in the Appendix). The IM questionnaire, adapted from Pratscher et al. (2019), was modified for age-appropriateness based on Maynard et al.’s (2017) guidelines. Preliminary testing indicated that children comprehended three dimensions: Presence (maintaining attention during social interactions, PR; 3 items), Awareness of Self and Others (recognition of personal and others’ states, AW; 5 items), and Nonreactivity (measured responses in social contexts, NR; 3 items). The dimension of Nonjudgmental Acceptance was excluded due to comprehension difficulties. The EI questionnaire, adapted from Wong and Law (2002), assessed through four items targeting emotion recognition and management. The MI questionnaire, based on Apaolaza et al. (2019), focused on attentional focus in academic contexts, using six items addressing sustained attention during classroom activities and homework. These instruments were chosen and adapted for their alignment with the study’s target constructs while ensuring developmental appropriateness for primary school children. Items were selected to maintain construct validity and comprehensibility, resulting in a 21-item instrument featuring a mix of positively and negatively phrased questions, presented in random order to reduce structural bias. Responses were recorded on a 6-point Likert scale.

Psychometric validation followed a multi-step process. The questionnaires were first translated from English to Chinese by two bilingual experts in mindfulness and child psychology, then back-translated to ensure conceptual equivalence. Content validity was assessed by three mindfulness practitioners and two child psychologists, with modifications made to enhance age-appropriateness for Taiwanese primary school students. Items were adapted to align with the cognitive and reading comprehension levels of children aged 10–13 years. Internal consistency was acceptable, with Cronbach’s *α* values of 0.708 (IM), 0.658 (EI), and 0.886 (MI). Preliminary testing with 32 students (grades 3–6) confirmed the clarity, comprehensibility, and cultural relevance of the Chinese versions. These students were excluded from the main study to prevent contamination. While initial psychometric analyses support the reliability and validity of the questionnaires, further validation—such as test–retest reliability and convergent validity—is recommended to strengthen their applicability in Taiwanese educational settings.

### Procedures

This study employed a cluster randomized design, with classes serving as the units of randomization. This design is well-suited for school-based interventions as it minimizes the risk of treatment contamination among students within the same class (Torgerson, 2008; Kriemler et al., 2010). The study was conducted from March to June 2024, with the MBP intervention delivered as a formal course for middle and senior-grade classes. The program was sponsored by WCGCTF, FPG (Taiwan), and all sessions were facilitated by Dr. Lin. The intervention was integrated into existing courses at Guanghua Elementary School (New Taipei, Taiwan) and adhered to CONSORT guidelines (Moher et al., 2010). To ensure allocation concealment, the school’s Academic Affairs Office generated the random allocation sequence using computer-generated random numbers in Microsoft Excel. The office also handled cluster enrollment and assigned classes to either the intervention or control groups. The research team was responsible for designing the study protocol and implementing the intervention. Students without parental consent for participation engaged in alternative activities during the MBP sessions. Throughout the implementation, children in both groups were unaware of the intervention status of the other group. Given the nature of mindfulness-based interventions, students and trainers were inherently aware of their participation. However, students were unaware of the intervention status of their peers in the other group, and teachers were not informed of their class’s assignment to either the intervention or control condition. To ensure objectivity in data collection and analysis, all assessments were conducted and analyzed by independent researchers who were not involved in the intervention delivery and remained blinded to group allocation.

The intervention group participated in four weekly 40-min MBP sessions, with assessments conducted at three time points: pre-test (start of week 1), first post-test (end of week 4, Stage 1), and second post-test (week 12, Stage 2), as illustrated in Figure 1. All assessments were administered in a paper-and-pencil format, with teaching assistants supervising the process and providing guidance on the testing procedure. The eight-week interval between the post-tests was designed to evaluate the retention effects of the intervention. Participants in the control group underwent identical assessments at the same time points, with both groups following the same course schedules throughout the 12-week study period, except for the implementation of the MBP sessions in the intervention group.

### Statistical analysis

Data analysis was conducted using SPSS 23.0 (IBM Corp., Armonk, NY, USA) with a significance level set at *α* = 0.05. Normality was verified using the Kolmogorov–Smirnov test, and homogeneity of variances was assessed with Levene’s test. The analysis proceeded in two stages. First, independent t-tests were used to compare baseline scores between the intervention and control groups. Second, to maximize precision in measuring intervention effects and avoid potential masking of interaction effects that might occur with three-level timepoint comparisons, change scores were calculated for each participant by subtracting their baseline scores from scores at both post-intervention (Stage 1) and 8-week follow-up (Stage 2), allowing each participant to serve as their own control. Using these change scores, a 3-way repeated measures analysis of variance (RM ANOVA) was conducted for each outcome measure (PR, AW, NR, EI, and MI), with gender (boy vs. girl) and grade (middle vs. senior) as between-subject factors, and stage (Stage 1 vs. Stage 2) as the within-subject factor.

Mauchly’s test was applied to evaluate sphericity (equal variances between all pairs of repeated measures). When sphericity was violated (*p* < 0.05), Greenhouse–Geisser corrections were applied to adjust the degrees of freedom, ensuring more conservative significance tests. Effect sizes were reported as partial η^2^ values, in accordance with Cohen (1988)'s guidelines. For significant interaction effects, independent t-tests were performed to explore subgroup differences.

## Results

**Figure 2:**
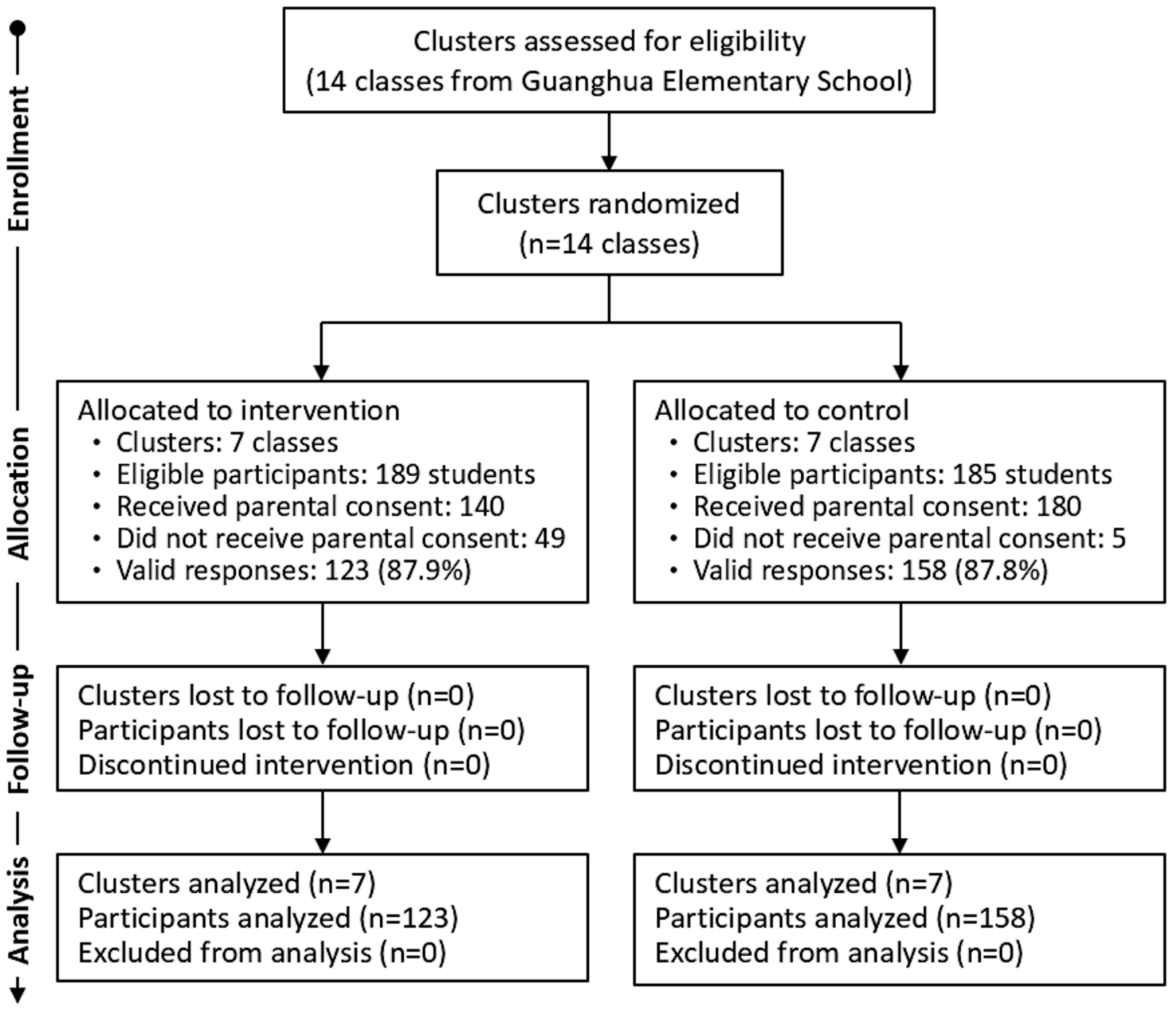
CONSORT flow diagram of the study, adapted from Moher et al. (2010).

Figure 2 illustrates the CONSORT flow diagram for this study. Of the 374 children initially randomized, 189 were allocated to the intervention group and 185 to the control group. While all participants agreed to join the study, parental consent rates differed (intervention: *n* = 140; control: *n* = 180), reflecting some parental hesitancy toward mindfulness practices. Valid responses totaled 281 (intervention: *n* = 123, 87.9%; control: *n* = 158, 87.8%), yielding an overall response rate of 88%. The mean age of children in both groups was similar: 12.1 years (range: 10–13) for the intervention group and 12.2 years (range: 10–13) for the control group. Figure 1 shows subgroup distributions across assessment stages.

Mauchly’s test indicated violations of sphericity for PR (*χ*^2^(1) = 8.45, *p* = 0.015), AW (*χ*^2^(1) = 7.92, *p* = 0.019), and NR (*χ*^2^(1) = 9.13, *p* = 0.010), necessitating Greenhouse–Geisser corrections (*ε* = 0.89, 0.91, and 0.87, respectively). In contrast, for EI (*χ*^2^(1) = 4.28, *p* = 0.118) and MI (*χ*^2^(1) = 5.13, *p* = 0.077), the sphericity assumption was met.

### Comparison of scores between groups

**Figure 3:**
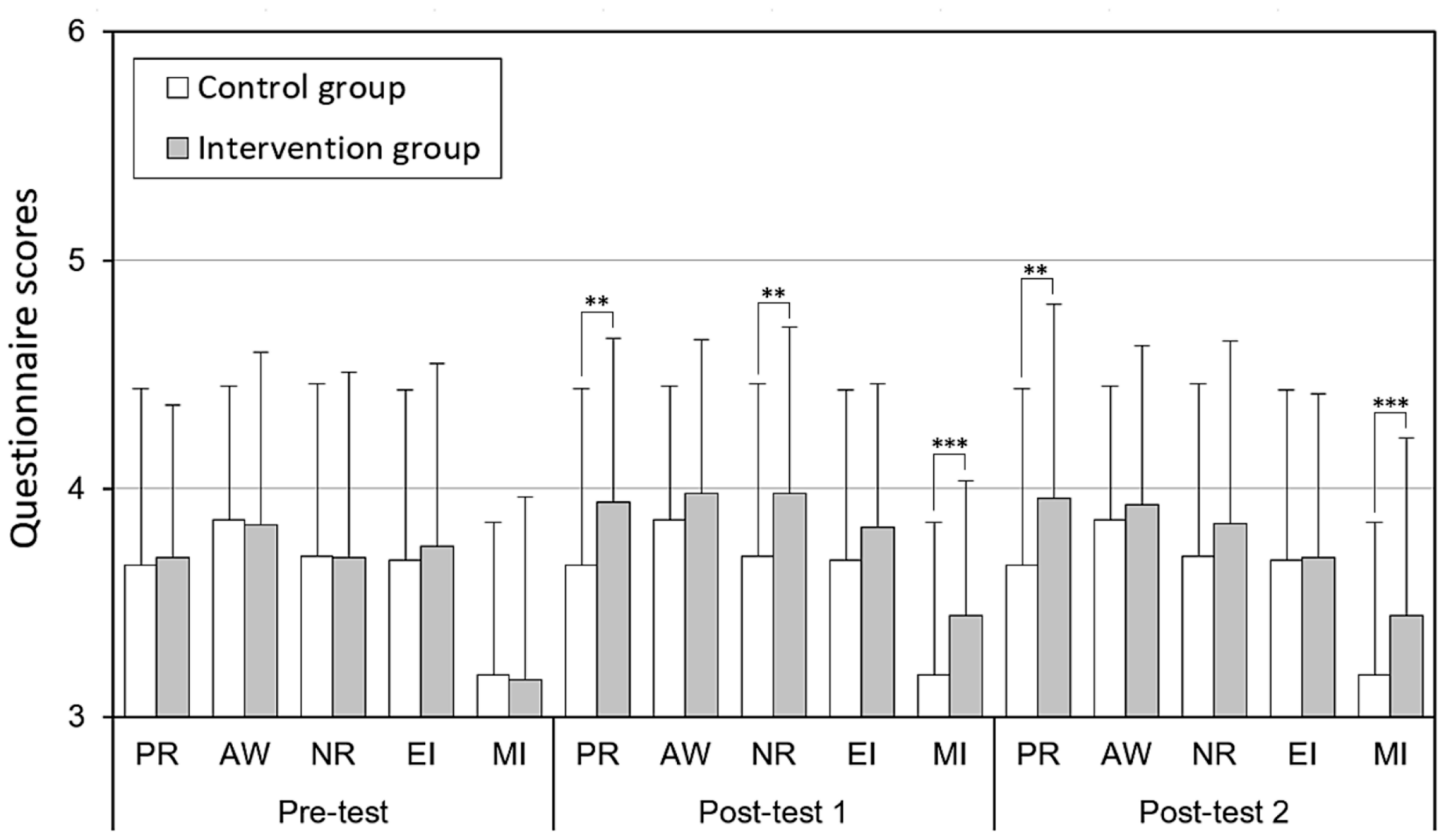
Comparison of the five dimensions (PR, AW, NR, EI, MI) between the intervention and control groups across three assessment time points (**p < 0.01, ***p < 0.001). PR = Presence; AW = Awareness; NR = Nonreactivity; EI = Emotional Intelligence; MI = Mindfulness.

The descriptive statistics for the outcome measures across all subgroups at the three time points are presented in Table 1. Independent t-tests confirmed no significant baseline differences between intervention and control groups across all five questionnaire dimensions (PR, AW, NR, EI, and MI) at pre-test, ensuring a valid basis for comparison (Figure 3). Following the MBP intervention, the first post-test revealed significantly higher scores in the intervention group for PR (*p* < 0.01), NR (*p* < 0.01), and MI (*p* < 0.001) compared to the control group. At the second post-test, conducted 8 weeks of post-intervention, the NR improvement was no longer evident, but significant gains in PR (*p* < 0.01) and MI (*p* < 0.001) persisted.

**Table 1:** Descriptive statistics of outcome measures across three time points by group, grade, and gender.

Measures	Group	Grade	Gender	*n*	Pre-test	Post-test 1	Post-test 2
Presence (PR)	Intervention	Middle	Boys	42	3.70 (0.67)	3.941 (0.72)	3.96 (0.83)
			Girls	46	3.74 (0.68)	4.12 (0.73)	4.10 (0.84)
		Senior	Boys	16	3.72 (0.80)	3.89 (0.79)	3.85 (0.87)
			Girls	19	3.66 (0.81)	3.80 (0.84)	3.78 (0.96)
	Control	Middle	Boys	58	3.66 (0.76)	3.45 (0.77)	3.68 (0.74)
			Girls	61	3.73 (0.78)	3.82 (0.78)	3.64 (0.77)
		Senior	Boys	21	3.71 (0.88)	3.58 (0.90)	3.76 (0.88)
			Girls	18	3.62 (0.88)	3.73 (0.84)	3.54 (0.81)
Awareness (AW)	Intervention	Middle	Boys	42	3.89 (0.74)	4.11 (0.65)	4.04 (0.67)
			Girls	46	3.77 (0.75)	3.75 (0.66)	3.76 (0.68)
		Senior	Boys	16	3.83 (0.82)	4.05 (0.88)	3.98 (0.87)
			Girls	19	3.86 (0.91)	3.84 (0.91)	3.85 (0.86)
	Control	Middle	Boys	58	3.91 (0.58)	3.87 (0.63)	3.88 (0.64)
			Girls	61	3.80 (0.59)	3.88 (0.66)	3.79 (0.73)
		Senior	Boys	21	3.88 (0.71)	3.84 (0.82)	3.89 (0.81)
			Girls	18	3.84 (0.78)	3.89 (0.70)	3.82 (0.73)
Nonreactivity (NR)	Intervention	Middle	Boys	42	3.63 (0.79)	3.93 (0.71)	3.88 (0.78)
			Girls	46	3.76 (0.80)	3.89 (0.72)	3.78 (0.80)
		Senior	Boys	16	3.69 (0.83)	3.98 (0.74)	3.75 (0.82)
			Girls	19	3.71 (0.88)	4.16 (0.85)	3.90 (0.90)
	Control	Middle	Boys	58	3.73 (0.72)	3.68 (0.76)	3.79 (0.82)
			Girls	61	3.67 (0.74)	3.72 (0.75)	3.64 (0.78)
		Senior	Boys	21	3.74 (0.80)	3.69 (0.85)	3.78 (0.83)
			Girls	18	3.68 (0.86)	3.73 (0.91)	3.65 (0.86)
Emotional Intelligence (EI)	Intervention	Middle	Boys	42	3.72 (0.78)	3.99 (0.61)	3.81 (0.70)
			Girls	46	3.80 (0.79)	3.81 (0.66)	3.78 (0.73)
		Senior	Boys	16	3.70 (0.82)	3.98 (0.75)	3.77 (0.74)
			Girls	19	3.74 (0.91)	4.12 (0.80)	3.91 (0.83)
	Control	Middle	Boys	58	3.67 (0.72)	3.72 (0.77)	3.64 (0.74)
			Girls	61	3.71 (0.73)	3.66 (0.74)	3.74 (0.68)
		Senior	Boys	21	3.69 (0.76)	3.77 (0.80)	3.64 (0.81)
			Girls	18	3.70 (0.84)	3.65 (0.83)	3.73 (0.85)
Mindfulness (MI)	Intervention	Middle	Boys	42	3.17 (0.78)	3.45 (0.57)	3.46 (0.76)
			Girls	46	3.13 (0.79)	3.41 (0.58)	3.42 (0.80)
		Senior	Boys	16	3.21 (0.82)	3.48 (0.71)	3.49 (0.79)
			Girls	19	3.15 (0.81)	3.43 (0.75)	3.44 (0.82)
	Control	Middle	Boys	58	3.22 (0.67)	3.17 (0.65)	3.25 (0.61)
			Girls	61	3.16 (0.65)	3.21 (0.69)	3.15 (0.66)
		Senior	Boys	21	3.20 (0.82)	3.16 (0.79)	3.22 (0.71)
			Girls	18	3.19 (0.75)	3.24 (0.77)	3.14 (0.77)

Data are presented as mean (standard deviation).

### Three-way ANOVA results

Following Greenhouse–Geisser corrections for PR, AW, and NR, a three-way RM ANOVA was conducted to examine the effects of gender, grade level, and test stage on the changes in scores across the five questionnaire dimensions between the two groups, as shown in Table 2. The analysis identified significant main effects of gender on AW scores, grade level on PR and EI scores, and test stage on NR and EI scores (all *p* < 0.05). Boys showed greater improvements in AW scores (+0.22) compared to girls (−0.02). Middle-grade students exhibited larger increases in PR scores (+0.35) than senior-grade students (+0.13), although senior-grade students had higher overall EI scores. Significant interactions between gender and grade level were observed for both NR and EI score changes (*p* < 0.05), underscoring the need for further cross-analyses to fully interpret these effects. In contrast, no significant interaction effects were found for PR, AW, and MI, confirming the main effects.

**Table 2:** Results of three-way ANOVA on score changes across five dimensions.

Sources	Aspects	SS	DF	MS	*F*	*p*-value	*η* ^2^
Gender	PR	0.327	0.89	0.367	0.612	0.524	0.002
	AW	2.433	0.91	2.673	5.892	<0.01	0.022
	NR	0.006	0.87	0.007	0.015	0.958	<0.001
	EI	0.658	1	0.658	1.504	0.221	0.006
	MI	0.813	1	0.813	1.689	0.195	0.007
Grade	PR	2.333	0.89	2.622	4.234	<0.05	0.015
	AW	0.681	0.91	0.748	1.823	0.182	0.006
	NR	0.578	0.87	0.664	1.247	0.285	0.004
	EI	2.170	1	2.170	4.961	<0.05	0.020
	MI	0.406	1	0.406	0.843	0.359	0.004
Stage	PR	0.082	0.89	0.092	0.157	0.812	0.001
	AW	0.326	0.91	0.358	0.892	0.412	0.003
	NR	1.793	0.87	2.061	3.524	<0.05	0.015
	EI	1.224	1	1.224	2.799	<0.05	0.014
	MI	0.008	1	0.008	0.017	0.896	<0.001
Gender × Grade	PR	0.006	0.89	0.007	0.012	0.967	<0.001
	AW	0.063	0.91	0.069	0.183	0.795	0.001
	NR	2.190	0.87	2.517	4.127	<0.05	0.016
	EI	2.229	1	2.229	5.096	<0.05	0.021
	MI	0.025	1	0.025	0.053	0.819	<0.001
Gender × Stage	PR	0.385	0.89	0.433	0.742	0.467	0.003
	AW	0.002	0.91	0.002	0.004	0.982	<0.001
	NR	0.316	0.87	0.363	0.684	0.486	0.002
	EI	0.072	1	0.072	0.164	0.686	0.001
	MI	0.081	1	0.081	0.167	0.683	0.001
Grade × Stage	PR	0.081	0.89	0.091	0.157	0.812	0.001
	AW	0.405	0.91	0.445	1.104	0.334	0.004
	NR	1.114	0.87	1.281	2.247	0.124	0.008
	EI	0.125	1	0.125	0.285	0.594	0.001
	MI	0.024	1	0.024	0.050	0.823	<0.001
Gender × Grade × Stage	PR	0.322	0.89	0.362	0.621	0.512	0.002
	AW	0.292	0.91	0.321	0.797	0.442	0.003
	NR	0.052	0.87	0.060	0.112	0.854	<0.001
	EI	0.086	1	0.086	0.198	0.657	0.001
	MI	0.008	1	0.008	0.017	0.898	<0.001

PR = Presence; AW = Awareness; NR = Nonreactivity; EI = Emotional intelligence; MI = Mindfulness. Greenhouse–Geisser corrections were applied for PR (sphericity estimate *ε* = 0.89), AW (*ε* = 0.91), and NR (*ε* = 0.87).

### Interaction effects of gender and grade on NR and EI score changes

**Figure 4:**
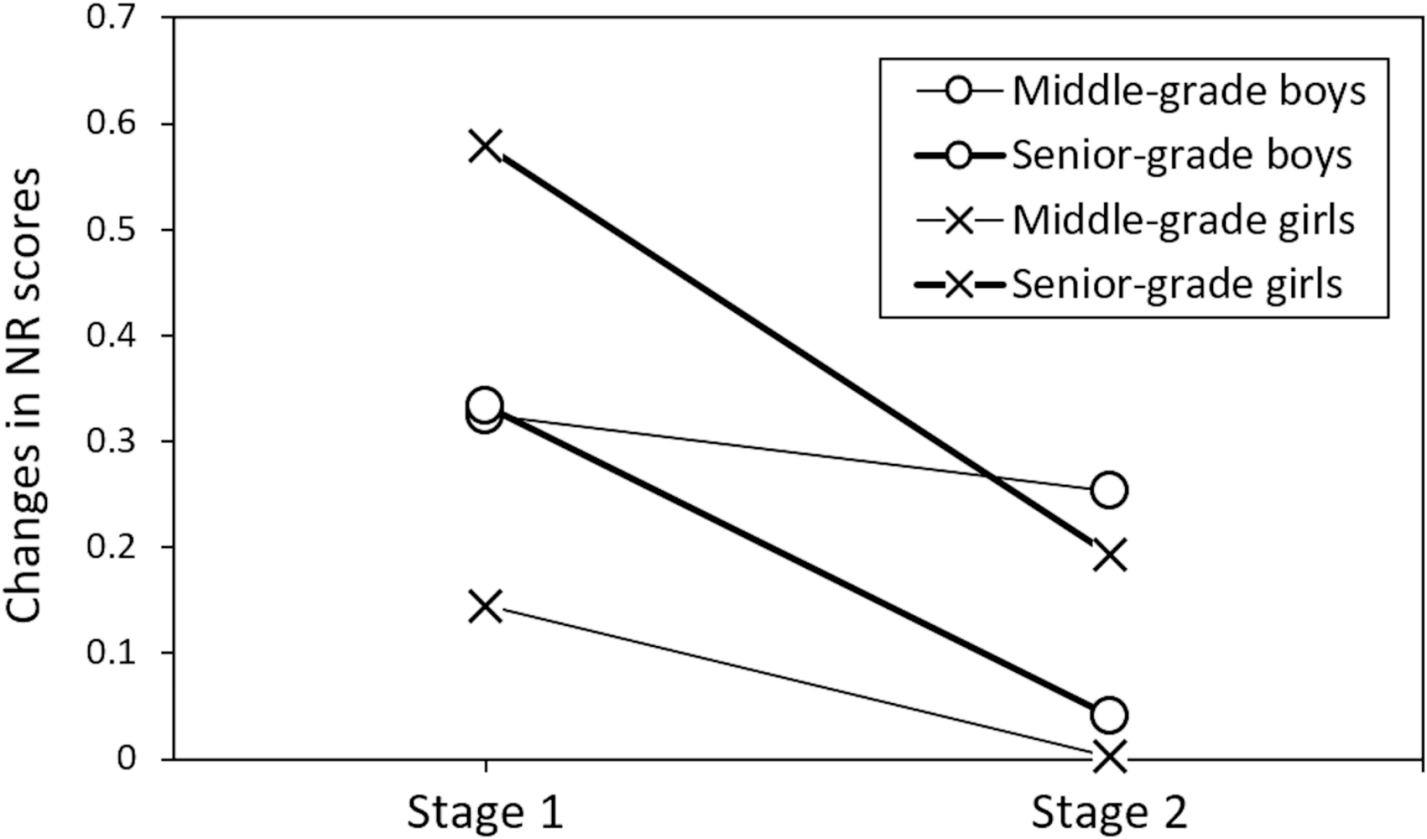
Interaction effects of gender and grade on Nonreactivity (NR) score changes across assessment stages.

**Figure 5:**
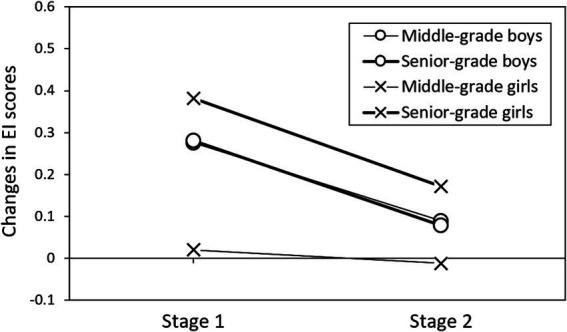
Interaction effects of gender and grade on Emotional intelligence (EI) score changes across assessment stages.

A cross-analysis of the interaction effects revealed distinct patterns for NR (Figure 4) and EI score changes (Figure 5). Additionally, the different stages were analyzed separately. For NR scores, both male and female senior-grade students exhibited significant decreases during stage 2, while middle-grade students showed more modest declines. Although *post-hoc* tests did not achieve statistical significance, these patterns suggest that the NR benefits of mindfulness programs may be most pronounced for senior-grade girls during the immediate intervention period (stage 1), with these improvements diminishing rapidly in the follow-up period (stage 2). EI score patterns varied by both gender and grade level. Male students consistently showed decreases in EI scores across both stages, irrespective of grade level. In contrast, female students demonstrated grade-dependent responses: middle-grade females showed no significant changes in EI scores, while senior-grade females exhibited significant improvements during stage 1, followed by a decline in stage 2.

## Discussion

This study employed a cluster randomized controlled trial to investigate the effects of a four-week MBP intervention on primary schoolchildren’s interpersonal mindfulness, emotional intelligence, and attentional focus. While broad improvements across all measured domains were hypothesized, the findings revealed nuanced patterns of effectiveness, with variations in both the magnitude and sustainability of improvements across dimensions. Notably, several observed results could not be directly compared to previous studies due to the limited research available on these specific topics, underscoring the need for further extensive and in-depth investigations.

The intervention group demonstrated significant improvements in PR, NR, and MI compared to the control group immediately following the intervention, consistent with recent research on the positive effects of short-term mindfulness programs on children’s emotional intelligence and attentional focus (Pickerell et al., 2023; Zhong et al., 2024). Notably, while PR and MI improvements persisted through the eight-week follow-up period, NR benefits diminished, indicating differential stability among mindfulness components. These findings align with prior studies suggesting that foundational mindfulness skills can develop through brief interventions, though the durability of effects may vary across specific domains (Lim et al., 2023; Dell’Aversana et al., 2023). Interestingly, MI scores (mean = 3.17) were significantly lower than other dimensions (mean = 3.74, Figure 3), highlighting low concentration levels among students in both groups during classroom activities and homework. This underscores the importance of further research on strategies to enhance children’s concentration through mindfulness interventions (Maynard et al., 2017; Carsley et al., 2018).

Gender-specific findings revealed notable patterns, particularly in AW scores, where boys demonstrated greater improvements. This aligns with developmental theories highlighting gender differences in social–emotional processing during primary school years (Yang et al., 2023; Denham et al., 2009). Boys’ enhanced AW gains may reflect differing developmental trajectories in emotional recognition and social awareness between genders (Wolf et al., 2021). Theoretical frameworks suggest that mindfulness interventions may particularly benefit male students’ interpersonal awareness development, given their typically later maturation in emotional recognition skills (Maynard et al., 2017).

Grade-level differences were consistent with cognitive developmental theory, with middle-grade students showing larger increases in PR scores. This supports models emphasizing heightened plasticity in mindfulness-related skills among younger children (Zelazo and Lyons, 2012). However, middle-grade students’ lower EI scores, despite higher PR scores, align with stage-sequential models of emotional development, which suggest that complex emotional intelligence skills develop independently and later than basic mindfulness capabilities (Costa and Faria, 2023; Malti and Noam, 2016).

Significant gender-grade interactions in NR and EI scores (Figures 4, 5) also align with developmental timing differences between boys and girls (Yang et al., 2023). Senior-grade students’ initial NR improvements, followed by declines, reflect developmental stage theories highlighting critical periods for intervention effectiveness (Maynard et al., 2017). Additionally, the grade-dependent EI patterns in female students—where senior-grade females showed improvements while middle-grade females demonstrated decreases—further support theoretical predictions about optimal timing for emotional intelligence interventions (Wang et al., 2023). These findings underscore the importance of tailoring mindfulness interventions to both gender and developmental stage, as suggested by Carsley et al. (2018).

Our findings on the relationship between mindfulness practice and academic focus reinforce the evidence that mindfulness interventions support broader educational objectives. The sustained improvements in attention and presence align with recent studies highlighting mindfulness’s positive effects on academic engagement and cognitive performance (Mora Álvarez et al., 2023; Zhong et al., 2024; Lin et al., 2024). These results suggest that mindfulness practices may serve as effective tools for enhancing both academic and social–emotional outcomes in primary education. The study’s implementation in Taiwan’s educational context revealed important cultural considerations. While mindfulness practices have Asian origins, their integration into modern educational systems necessitates careful adaptation to local contexts (Blignault et al., 2023; Haidar et al., 2024). This was particularly evident in the disparity of parental consent rates between intervention (74.1%) and control (97.3%) groups, reflecting varying cultural attitudes toward mindfulness practices in educational settings.

Beyond our primary objectives, several unexpected effects emerged that merit attention. We observed notable gender differences, with boys showing greater improvements in AW scores compared to girls, contrasting with previous findings that suggested girls typically demonstrate better mindfulness outcomes (Kang et al., 2018; Rojiani et al., 2017). Additionally, grade-level differences revealed that while middle-grade students demonstrated larger increases in PR scores than senior-grade students, senior-grade students showed better performance in emotional intelligence, aligning with developmental theories of emotional maturity (Denham et al., 2009; Malti and Noam, 2016). These findings offer valuable insights for future program implementation and underscore the importance of considering demographic factors when designing mindfulness interventions for primary school students (Maynard et al., 2017; Carsley et al., 2018).

Beyond our primary objectives, several unexpected effects and safety considerations merit attention. We observed notable gender differences, with boys showing greater improvements in AW scores compared to girls, contrasting with previous findings that suggested girls typically demonstrate better mindfulness outcomes (Kang et al., 2018; Rojiani et al., 2017). Additionally, grade-level differences revealed that while middle-grade students demonstrated larger increases in PR scores than senior-grade students, senior-grade students showed better performance in emotional intelligence, aligning with developmental theories of emotional maturity (Denham et al., 2009; Malti and Noam, 2016).

No adverse events or unintended negative effects were reported by students, teachers, or parents throughout the intervention. Some students initially experienced mild difficulty maintaining attention during mindfulness exercises, but these challenges were transient and resolved with practice. Teaching assistants supervising the sessions observed no concerning behaviors or negative reactions, and no parents expressed concerns about their children’s participation. This aligns with previous research indicating that age-appropriate mindfulness programs are generally well-tolerated by primary school children (Porter et al., 2022; Kander et al., 2024). However, the absence of a formal adverse event monitoring system in this study highlights the need for future research to systematically track potential unintended effects.

Several limitations of this study warrant consideration. While the four-week intervention period facilitated practical implementation, it may have been too brief to fully capture potential benefits, particularly in complex domains such as emotional intelligence. A key methodological limitation is the small number of clusters (14 classes, restricted by the scope of the school), which falls below the recommended minimum of 30 clusters for robust multilevel statistical analyses (McNeish and Stapleton, 2016). This constraint precluded the consideration of classroom clustering effects, such as inter-cluster correlations and cross-level interactions. Additionally, the absence of *a priori* clinical trial registration raises concerns about potential selective outcome reporting. Methodological constraints also include the reliance on self-report measures, which may introduce response bias, particularly among younger participants. The lack of physiological or behavioral measures further limited the validation of findings through objective indicators. Although pilot testing and refinement of questionnaire items aimed to ensure comprehensibility, variations in language development among primary school students may have affected question interpretation, a key consideration for mindfulness interventions at this developmental stage (Maynard et al., 2017; Carsley et al., 2018). The absence of active control group activities and the specific Taiwanese cultural context may also limit the generalizability of the findings.

Future research should address these limitations by incorporating larger cluster samples with appropriate multilevel analytical methods, extending intervention periods, implementing objective measures, and conducting cross-cultural validation studies. Priority should be given to elucidating the mechanisms underlying gender-and age-related differences in intervention effectiveness and developing strategies to sustain improvements in areas where benefits diminish over time. Further exploration of family factors, prior meditation experience, and cultural attitudes toward mindfulness could provide valuable insights for optimizing program design and implementation across diverse student populations.

## Conclusion

This study demonstrates that a four-week MBP intervention can effectively enhance specific aspects of interpersonal mindfulness and attentional focus among primary school children, with select benefits persisting through an eight-week follow-up. The findings underscore significant grade-level and gender differences in response to mindfulness training, highlighting the importance of tailored interventions that address these demographic factors. While improvements in presence and general mindfulness exhibited sustained effects, the transient nature of other benefits, particularly in nonreactivity, suggests the need for ongoing practice or booster sessions to maintain comprehensive outcomes.

These results hold important implications for educational practice and research, indicating that even brief mindfulness interventions, when developmentally and culturally adapted, can yield meaningful benefits. This study contributes to the growing body of evidence supporting the integration of mindfulness into primary education while acknowledging the complexities of implementation across diverse student populations. Future research should focus on optimizing intervention duration, developing strategies for long-term benefit maintenance, and designing targeted approaches for different student subgroups. Such efforts will be instrumental in advancing the development of more effective and sustainable mindfulness education programs in school settings.

## Data Availability

The raw data supporting the conclusions of this article will be made available by the authors, without undue reservation.

## Appendix

The questionnaire items used in this study are listed below. Items marked with (―) indicate reverse-scored items, where higher scores reflect lower levels of the measured construct.

1. Interpersonal mindfulness (IM)
(1) Presence (PR)

- When I am with others, I am easily distracted. (―)
- When a person is talking to me, I do not find myself thinking about other things.
- When I am conversing with another person, I am fully engaged in the conversation.
- When speaking to another person, I am aware of how I feel inside.

(2) Awareness of Self and Others (AW)

- I maintain eye contact with the person I’m interacting with.
- I can perceive others’ emotional states during interactions.
- I am aware of my facial and body expressions when interacting with others.
- Supporting others brings me joy.
- When I offer encouragement to others, I feel uplifted as well.

(3) Nonreactivity (NR)

- I take time to form my thoughts before speaking.
- I practice attentive and patient listening when others speak.
- I allow others to complete their thoughts without interruption.

2. Emotional intelligence (EI)

- I have good understanding of my own emotions.
- I can always calm down quickly when I am very angry.
- I can accept my emotional experiences.
- I can conveyor my current emotional state.

3. Mindfulness (MI)

- When I am in classroom activities, I’m easily distracted. (―)
- When I am doing homework, I’m easily distracted. (―)
- I can maintain sustained attention during classroom activities.
- I can maintain focused concentration while completing homework.
- I find myself in classroom activities without paying attention. (―)
- I find myself doing homework without paying attention. (―)
